# The 26S Proteasome Regulatory Subunit GmPSMD Promotes Resistance to *Phytophthora sojae* in Soybean

**DOI:** 10.3389/fpls.2021.513388

**Published:** 2021-01-28

**Authors:** Tengfei Liu, Huiyu Wang, Zhanyu Liu, Ze Pang, Chuanzhong Zhang, Ming Zhao, Bin Ning, Bo Song, Shanshan Liu, Zili He, Wanling Wei, Junjiang Wu, Yaguang Liu, Pengfei Xu, Shuzhen Zhang

**Affiliations:** ^1^Key Laboratory of Soybean Biology of Chinese Education Ministry, Soybean Research Institute, Northeast Agricultural University, Harbin, China; ^2^Key Laboratory of Soybean Cultivation of Ministry of Agriculture P. R. China, Soybean Research Institute of Heilongjiang Academy of Agricultural Sciences, Harbin, China

**Keywords:** soybean, *Phytophthora sojae*, *GmPIB1*, *GmPSMD*, ROS

## Abstract

Phytophthora root rot, caused by *Phytophthora sojae* is a destructive disease of soybean (*Glycine max*) worldwide. We previously confirmed that the bHLH transcription factor GmPIB1 (*P. sojae*-inducible bHLH transcription factor) reduces accumulation of reactive oxygen species (ROS) in cells by inhibiting expression of the peroxidase-related gene *GmSPOD* thus improving the resistance of hairy roots to *P. sojae*. To identify proteins interacting with GmPIB1 and assess their participation in the defense response to *P. sojae*, we obtained transgenic soybean hairy roots overexpressing GmPIB1 by *Agrobacterium rhizogenes* mediated transformation and examined GmPIB1 protein–protein interactions using immunoprecipitation combined with mass spectrometry. We identified 392 proteins likely interacting with GmPIB1 and selected 20 candidate genes, and only 26S proteasome regulatory subunit GmPSMD (Genbank accession no. XP_014631720) interacted with GmPIB1 in luciferase complementation and pull-down experiments and yeast two-hybrid assays. Overexpression of *GmPSMD* (*GmPSMD-*OE) in soybean hairy roots remarkably improved resistance to *P. sojae* and RNA interference of *GmPSMD* (*GmPSMD* -RNAi) increased susceptibility. In addition, accumulation of total ROS and hydrogen peroxide (H_2_O_2_) in *GmPSMD-*OE transgenic soybean hairy roots were remarkably lower than those of the control after *P. sojae* infection. Moreover, in *GmPSMD*-RNAi transgenic soybean hairy roots, H_2_O_2_ and the accumulation of total ROS exceeded those of the control. There was no obvious difference in superoxide anion (O_2_^–^) content between control and transgenic hairy roots. Antioxidant enzymes include peroxidase (POD), glutathione peroxidase (GPX), superoxide dismutase (SOD), catalase (CAT) are responsible for ROS scavenging in soybean. The activities of these antioxidant enzymes were remarkably higher in *GmPSMD*-OE transgenic soybean hairy roots than those in control, but were reduced in *GmPSMD*-RNAi transgenic soybean hairy roots. Moreover, the activity of 26S proteasome in *GmPSMD*-OE and *GmPIB1*-OE transgenic soybean hairy roots was significantly higher than that in control and was significantly lower in *PSMD*-RNAi soybean hairy roots after *P. sojae* infection. These data suggest that *GmPSMD* might reduce the production of ROS by improving the activity of antioxidant enzymes such as POD, SOD, GPX, CAT, and *GmPSMD* plays a significant role in the response of soybean to *P. sojae*. Our study reveals a valuable mechanism for regulation of the pathogen response by the 26S proteasome in soybean.

## Introduction

Phytophthora root rot is caused by the oomycete pathogen *Phytophthora sojae* Kaufmann and Gerdemann and destroys soybean crops worldwide (Schmitthenner, 1972, 1985; Tyler, 2007). Breeding resistant cultivars is an effective and economical measure to control this disease; however, rapid changes in the pathogen population quickly overcome the resistance of new cultivars (Schmitthenner et al., 1996). Therefore, isolating resistance-related genes and studying resistance mechanisms has great potential value for improving soybean disease resistance through genetic engineering.

Protein homeostasis facilitates cell senescence and protects cells from disease. As the main cellular protease complex, the 26S proteasome is at the core of maintaining protein homeostasis in cells (Smalle and Vierstra, 2004; Kurepa and Smalle, 2008). The 26S proteasome can be divided into two subcomplexes: the core particle (20S) and the regulatory particle (19S). The 19S regulatory particle consists of at least 19 proteins and the 20S core particle consists of 28 proteins. The difference between 20S core particles and 19S regulatory particles is that 20S core particles can degrade proteins without hydrolyzing ATP (Kish-Trier and Hill, 2013), and they cannot recognize and degrade protein substrates labeled by ubiquitin chains (Stadtmueller et al., 2011; Bhattacharyya et al., 2014). The 19S regulatory particle acts as a receptor, assisting in ubiquitination and unfolding of ubiquitinated protein substrates. Multiple catalytic sites degrade the substrate into short polypeptides, which are subsequently broken down by peptidases into peptides and amino acids that are recycled by cells (Tanaka et al., 1986; Hough et al., 1987).

The ubiquitin/26S proteasome pathway is widely involved in the regulation of plant development and growth (Silverstone et al., 2001; Tyler, 2004; Yu et al., 2015), signal transduction (Itoh et al., 2002; Potuschak et al., 2003; Smalle et al., 2003; Dill, 2004; Fleet and Sun., 2005), ATP-dependent degradation of ubiquitin (Clough et al., 1999; Hardtke et al., 2000; Schultz, 2001; Schwechheimer and Deng, 2001), and plant responses to biotic and abiotic stresses (Silverstone et al., 2001; Tyler, 2004; Fleet and Sun, 2005). A large body of evidence indicates that the 26S proteasome is involved in the defense response of cells to pathogens. Some 26S proteasome subunits interact with pathogens effectors to inhibit their own activity and trigger PAMP-triggered immunity (PTI) and Effector-triggered Immunity (ETI) responses (Park et al., 2012; Üstün et al., 2014). For example, the effector protein AvrPiz-t of *Magnaporthe grisea* is degraded by the ubiquitin/proteasome pathway, which regulates PTI responses (Park et al., 2012). Moreover, effector proteins from the pathogen can target the proteasome. Üstün et al. (2014) reported that the 26S proteasome subunit RPT6 interacts with the effector protein HopZ4, which inhibits the activity of the 26S proteasome making plants more susceptible to infection. In Arabidopsis, RPM1, a peripheral membrane protein mediates a hypersensitive response, and induction of RPM1 can increase plant defenses. Pathogen invasion shortens the half-life of RPM1, indicating that the ubiquitin/26S proteasome pathway is involved in defense against pathogens (Qiao et al., 2004). The yeast protein SGT1 was originally identified as a defense-related protein that could associate with SKP1 and CUL1, subunits of the Skp1-Cullin–F-box ubiquitin ligase complex (SCF) (Boyes et al., 1998). The orthologous plant protein SGT1 is also involved in the early defense response against pathogens, and entry of a pathogen induces attachment of the SGT1-SCF complex to the target protein, mediating its connection and degradation in Arabidopsis (Azevedo et al., 2002).

Programmed cell death plays a critical role in plant disease resistance and is triggered by resistance (R) proteins (Coll et al., 2011). Vacuoles and plasma membranes mediated by the PBA1 subunit of the 26S proteasome fuse together to release antimicrobial substances (Hatsugai et al., 2015). The Rho GTPase-activating protein SPIN6 interacted with U-box E3 ligase SPIN11 is also a key factor in the rice defense response (Liu et al., 2015). SPL11 promotes the ubiquitination of SPIN6, and *SPIN6*-RNAi plants show enhanced resistance to *Xanthomonas oryzae* and *Magnaporthe oryzae* (Liu et al., 2015). Arabidopsis botrytis susceptible1 (BO1) interacts with the MYB transcription factor BOS1, involved in the Arabidopsis defense response (Luo et al., 2010). BO1 can also ubiquitinate BOS1, and both BOS1 and its homologous genes can resist to the saprophytic fungus *Botrytis cinerea* in Arabidopsis (Luo et al., 2010). Since the 26S proteasome system plays a crucial regulatory role in plant defense responses, it is a target for utilization by pathogens (Marino et al., 2012). The 26S proteasome 19S regulatory subunit RPN6 interacts with XopJ, a type III effector protein of *Xanthomonas*. RPN6 is induced by XopJ and transferred to the cell membrane. XopJ has protease activity to specifically degrade RPT6, leading to reduced proteasome activity. Inhibition of 26S proteasome leads to abnormal vesicle trafficking, callus reduction, and salicylic acid (SA) signal transduction (Üstün et al., 2013; Üstün and Börnke, 2015).

Reactive oxygen species (ROS) play a key role in plant disease resistance. It is obvious that chemical substances such as ammonium diphenyliodonium chloride inhibit the accumulation of ROS during attacking by microbial pathogens, and ammonium diphenyliodochloride is thought to inhibit the accumulation of ROS-producing NADPH oxidase (Jabs et al., 1997). Moreover, ROS-producing systems triggered plant defense mechanism in plant (Wu et al., 1997). In our previous study, the bHLH transcription factor GmPIB1 (*P. sojae*-inducible bHLH transcription factor) reduces the accumulation of ROS in cells by inhibiting expression of the peroxidase-related gene *GmSPOD*, thus improving the resistance of soybean hairy roots to *P. sojae* (Cheng et al., 2018). Here, we identified proteins interacting with GmPIB1 and tested whether they participate in the defense response to *P. sojae*, finding that the 26S proteasome regulatory subunit GmPSMD interacts with GmPIB1. Overexpressing *GmPSMD* improved the activity of antioxidant enzymes simultaneously decreased ROS accumulation and enhanced resistance to *P. sojae*; conversely, knocking down *GmPSMD* increased susceptibility, and ROS accumulation and antioxidant enzymes activity was inhibited. These results indicated that *GmPSMD* improves the activity of antioxidant enzymes and suppresses ROS accumulation, suggesting that *GmPSMD* plays a positive regulatory role in the response of soybean to *P. sojae*.

## Materials and Methods

### Plant Materials and Growth Conditions

The *P sojae*-susceptible soybean cultivar “Dongnong 50” and the resistant cultivar “Suinong10” were used. Seeds of the resistant cultivar “Suinong 10” were grown in an incubator at 25°C, 70% relative humidity under a 16 h light/8 h dark cycle, and the seedlings at stage V1 (the first true leaves were about to unfold; Fehr et al., 1971) were inoculated with *P. sojae* zoospores according to the methods described by Ward et al. (1979) and Yang et al. (1996) with minor modifications. Seeds of the susceptible cultivar “Dongnong 50” were obtained from the Key Laboratory of Soybean Biology in the Chinese Ministry of Education, Harbin, and this cultivar was used for genetic transformation experiments.

### Bioinformatics Analysis of GmPSMD

The online database NCBI (National Center for Biotechnology Information) was used for finding the sequences of high homology genes with GmPSMD. DNAMAN software^1^ was used for the sequence multiple alignments, and a phylogenetic analysis of GmPSMD was carried out using MEGA software. The GmPSMD protein structure was predicted using Phyre2^2^.

### RT-PCR and qRT-PCR Analysis

Total RNA was isolated using Trizol reagent and reverse-transcribed based on the manufacturer’s instructions (Invitrogen, China). cDNA was synthesized from 1 μg of total RNA using a Super Script first-strand cDNA synthesis system (Takara, Dalian, China). qRT-PCR was performed on a LightCycler96 instrument (Roche, Switzerland) using a real-time PCR kit (ToYoBo, Japan). The soybean *GmEF1*β housekeeping gene (GenBank accession no. NM_001248778) was used as an internal control to normalize all data (Supplementary Table S1). The relative transcript level of target genes was calculated using the 2^–^^Δ^^Δ^^*CT*^ method. Three biological repeats for each line were performed in each experiment.

### Yeast Two-Hybrid Assays

Yeast two-hybrid assays were performed using the Frozen-EZ Yeast Transformation II kit (The Epigenetics Company, United States). The full-length *GmPSMD* was inserted into the pGADT7 expression vector, and *GmPIB1* was inserted into the pGBKT7 expression vector. Fusion plasmids pGADT7-GmPSMD and pGBKT7-GmPIB1 were transformed into yeast strain Y_2_HGold (Takara Bio, Japan) to identify protein–protein interactions. pGBKT7-53 and pGADT7-SV40 plasmids were used as a positive control, while pGBKT7-Lam and pGADT7-SV40 plasmids were used as the negative control in yeast cells.

### Luciferase Complementation Assays

To construct *GmPIB1-ccluc* and *GmPSMD-nluc*, the coding sequences of *GmPIB1* and *GmPSMD* were cloned into the plant expression vectors pCAMBIA1300-ccluc and pCAMBIA1300-nluc using gene-specific primers, respectively (Supplementary Table S1). The recombinant plasmids were transferred into *Agrobacterium tumefaciens GV3101*, and transformed Agrobacterium strains resistant to kanamycin and rifampicin were selected and cultured in YEP medium (containing 0.5% Yeast, 1% Peptone, 0.5% NaCl) at 28°C until the OD_600_ reached 0.8–1.2. Agrobacterium cells were collected by centrifugation at 4,000 rpm for 10 min, resuspended in infection liquid (containing 10 mM MgCl_2_, 10 mm MES, 150 μmol acetosyringone, pH 5.6) and placed at room temperature (25°C) for 2–3 h. The two recombinant plasmids were mixed in equal volumes and injected into *Nicotiana benthamiana*. Fluorescence signals were observed after dark culture for 2 days.

### Induction and Purification of Fusion Proteins

The open reading frames of *GmPSMD* and *GmPIB1* were, respectively, fused to the GST-tag of vector PGEX-4T-1 and the 6 × His-tag of vector pET29b (+) (Novagen, Germany) and then transformed into *Trans*etta (DE3) *Escherichia coli* cells (TransGen Biotech, China). GST-tagged and His-tagged proteins were induced with 0.5 mM isopropyl-β-D-thiogalactoside (IPTG) at 37°C for 4 h. GmPIB1-His and GmPSMD-GST fusion proteins were purified using a GST-Sefinose kit (Sangon, China) or a His-bind Purification Kit (Merck Millipore) at 4°C and subsequently detected by sodium dodecyl sulfate polyacrylamide gel electrophoresis (SDS-PAGE) and immunoblotting using anti-His and anti-GST antibodies (Abmart, United States), respectively.

### GST Pull-Down Assays

The GST fusion protein was fused with 50 μL GST beads (Sangon, China) at 4°C for 1 h then washed three times with 1 × phosphate-buffered saline (PBS) containing 1% Triton-100 before adding the purified fusion protein with His-tag and incubating at 4°C for 4 h. The washing step was repeated, and 40 μL 5 × loading buffer was added for immunoblotting detection.

### *Agrobacterium rhizogenes*-Mediated Transformation of Soybean Hairy Roots

To construct the pCAMBIA3301*-GmPIB1* and pCAMBIA3301-*GmPSMD* overexpression vector, the coding sequence of *GmPIB1* and *GmPSMD* was cloned into the plant expression vector pCAMBIA3301 with a C-terminal 4 × Myc fusion sequence, respectively. For RNA interference performance, the specific *GmPSMD* cDNA fragment was amplified and inserted into the vector PFGC5941 (Kerschen et al., 2004). “Dongnong 50” was used to generate transgenic soybean hairy roots by *A. rhizogenes*-mediated transformation following the instructions described by Paz et al. (2004). Transgenic soybean hairy roots were preliminarily detected by PCR (Primers shown in Supplementary Table S1), and then the level of gene overexpression or silencing were detected by qRT-PCR and those overexpressing GmPIB1 and *GmPSMD* were identified by immunoblotting with an anti-Myc antibody (Abmart, United States).

### Pathogen Response Assays of Transgenic Soybean Hairy Roots

Resistance to *P. sojae* was assessed based on methods described by Ward et al. (1979) with minor modifications. *GmPSMD*-transformed “Dongnong 50” soybean hairy roots were cultured for about 2 weeks and were placed on a tray with clean and moist gauze. All hairy roots were slightly scratched at the same position with a sterile scalpel, and zoospores of *P. sojae* race 1 were used to inoculate the wounds. Empty vector (EV) soybean hairy roots were used as controls. Disease symptoms were recorded by photography. The *P. sojae* biomass was analyzed based on the accumulation of *P. sojae TEF1* (GenBank accession no. EU079791), *PSEL1* (GenBank accession no. CF840149), and *PSEL2* (GenBank accession no. CF839332) in the transgenic soybean hairy roots. The pathogen response assays were performed on three biological replicates, each with three technical replicates.

### Detection of Total ROS and Antioxidant Enzyme Activity

Total ROS production was determined according to the instructions of Reactive Oxygen Species Assay Kit (Beyotime Institute of Biotechnology, China). Fluorescence was detected at 530 nm emission wavelength and 485 nm excitation wavelength using a Microplate Reader (Bio-TEK, United States; Qian et al., 2009). For the enzyme assays, 0.2 g of soybean transgenic hairy roots was ground with 25 mM HEPES buffer (pH 7.8) containing 0.2 mM EDTA, 2 mM ascorbate and 2% PVP. The homogenate was centrifuged at 4°C for 15 min at 12,000 × g and the resulting supernatant was used for the determination of the enzymatic activity following the method described by Cao et al. (2009). The superoxide dismutase (SOD) activity was measured as the increase in the absorbance at 560 nm according to instructions of Superoxide Dismutase Assay Kit (COMIN, Institute of Biotechnology, Suzhou of China). The catalase (CAT) activity was measured as the decline in the absorbance at 240 nm due to the decrease of extinction according to the instructions of Superoxide Dismutase Assay Kit (COMIN, Institute of Biotechnology, Suzhou of China). The guaiacol peroxidase (GPX) activity was measured as the increase in the absorbance at 340 nm due to guaiacol oxidation according to the instructions of Superoxide Dismutase Assay Kit (COMIN, Institute of Biotechnology, Suzhou of China). The peroxidase (POD) activity was measured as the increase in the absorbance at 470 nm according to the instructions of Superoxide Dismutase Assay Kit (COMIN, Institute of Biotechnology, Suzhou of China). The detection of total ROS and antioxidant enzyme activity assays were performed on three biological replicates, each with three technical replicates.

### Detection of H_2_O_2_ and O_2_^–^

Hydrogen peroxide (H_2_O_2_) accumulation was determined according to the method of Velikova et al. (2000). Superoxide anion (O_2_^–^) accumulation in *GmPSMD*-transformed and EV soybean hairy roots was determined as follows: soybean hairy roots were inoculated with *P. sojae* to induce production of O_2_^–^, and samples were taken at 0, 12, and 24 h. Then the samples were thoroughly ground and vortexed, and 500 μL of 10 mM phosphate buffer (PH = 7.8) was added. After centrifuging at 4°C and 5,000 rpm for 10 min, the supernatant was added with 700 μL phosphate buffer and 100 μL of 10 mM hydroxylamine hydrochloride at 25°C for 20 min. Finally, 0.5 mL of P-aminobenzenesulfonic acid and 0.5 mL of naphthylamine were added and reacted at 25°C for 20 min to read the absorbance of the supernatant at 530 nm.

### Determination of 26S Proteasome Activity

The 26S proteasome activity assays were performed as previously described by Kisselev and Goldberg (2005). Briefly, GmPSMD and GmPIB1 transgenic soybean hairy roots were inoculated with *P. sojae* and the samples were taken at 0, 12, and 24 h. The proteasome activity buffer 50 Mm Tris-HCl (pH = 7.5), 250 mM sucrose, 5 mM MgCl_2_, 0.5 mM EDTA, 2 mM ATP, 1 mM DTT, 1% of the total volume of PSMF and Roche inhibitor were prepared and added to the sample in proportion. The homogenate was centrifuged at 4°C for 15 min at 10,000 × g and the resulting supernatant was used for the determination of 26S proteasome activity. The supernatant, buffer and fluorescent substrate (proteasome substrate Ø, fluorogenic) were added to a 96-well plate (BD Flacon) at a ratio of 10:137.5:2.5. Fluorescence was detected at 460 nm emission wavelength and 380 nm excitation wave length at 25°C. The changes of fluorescence units in 10 min were recorded.

## Results

### Screening and Identification of GmPIB1-Interacting Proteins

**FIGURE 1:**
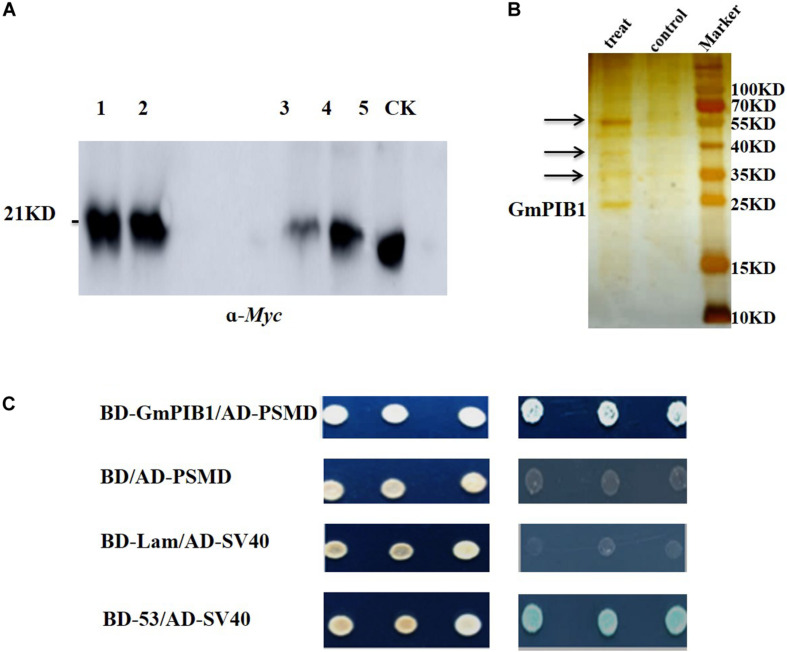
Screening and identification of GmPIB1-interacting proteins. **(A)** Immunoprecipitation experiment. Lanes 1 and 2, protein supernatant from positive hairy roots; lane 3, immunoprecipitated protein supernatant from the experimental group; lane 4, immunoprecipitated protein supernatant from the control group; lane 5, experimental group immunoprecipitation products; CK, control group immunoprecipitation products. **(B)** Silver nitrate staining. Treat: immunoprecipitated products containing GmPIB1-Myc protein with anti-Myc; control: immunoprecipitated products containing GmPIB1-Myc protein with negative antibody (anti-Mouse); arrows indicate different bands. **(C)** Interaction of GmPIB1 with GmPSMD in yeast cells. Yeast strain Y_2_HGold transformed with fusion plasmids pGADT7-GmPSMD and pGBKT7-GmPIB1 was grown on SD/-Trp/-Leu media and SD/-Trp/-Leu/-His/-Ade media with added X-α-gal. pGBKT7-53 and pGADT7-SV40 plasmids were used as a positive control; pGBKT7-Lam and pGADT7-SV40 plasmids were used as a negative control.

To explore the proteins interacting with GmPIB1, the transgenic soybean hairy roots overexpressing GmPIB1 were obtained using *Agrobacterium rhizogenes*-mediated transformation. Proteins interacting with GmPIB1 were screened by immunoprecipitation-mass spectrometry and verified by yeast two-hybrid library screening. pCAMBIA3301-*GmPIB1-myc* recombinant vector was successfully constructed (Supplementary Figure S1A) and the immunoblotting analysis confirmed successful production of GmPIB1 in transgenic soybean hairy roots (Supplementary Figure S1B). Then the immunoprecipitation was conducted and the specific fluorescent signal was observed in positive hairy roots (Figure 1A), indicating that protein activity of GmPIB1 was good. Specific stripes for co-immunoprecipitating proteins were observed in the experimental group, showing that the precipitation complex contained not only the target protein GmPIB1, but also proteins that may interact with GmPIB1. Several stripes observed in the experimental group and not in the control by silver staining (Figure 1B) were removed for mass spectrometry. completed by Wuhan GenecreateBiological Engineering Company. A total of 392 proteins that might interact with GmPIB1 were retrievaled and analyzed by Proteinpilot software, which were mainly related to energy metabolism, gene expression regulation, and transportion. Among the 392 proteins, 20 candidate genes were selected based on the identification score of mass spectrometry data (Supplementary Table S2). To verify interactions between proteins encoded by the candidate genes and GmPIB1, the full-length *GmPIB1* gene was inserted into vector pGBKT7 and the candidate genes into vector pGADT7. Recombinant vectors were transformed into yeast strain Y_2_H Gold and titrated on SD/-Leu/-Trp and SD/-Ade/-His/-Leu/-Trp plates to verify protein–protein interactions. Only yeast co-transformed with pGADT7-284 (*GmPSMD*) and pGBKT7-GmPIB1 grew on SD/-Ade/-His/-Leu/-Trp plates (Supplementary Figure S2). Moreover, GmPSMD had no self-activating activity and the yeast two-hybrid results show that GmPSMD and GmPIB1 interact in yeast (Figure 1C).

### GmPIB1 and GmPSMD Interact *in vitro* and *in vivo*

**FIGURE 2:**
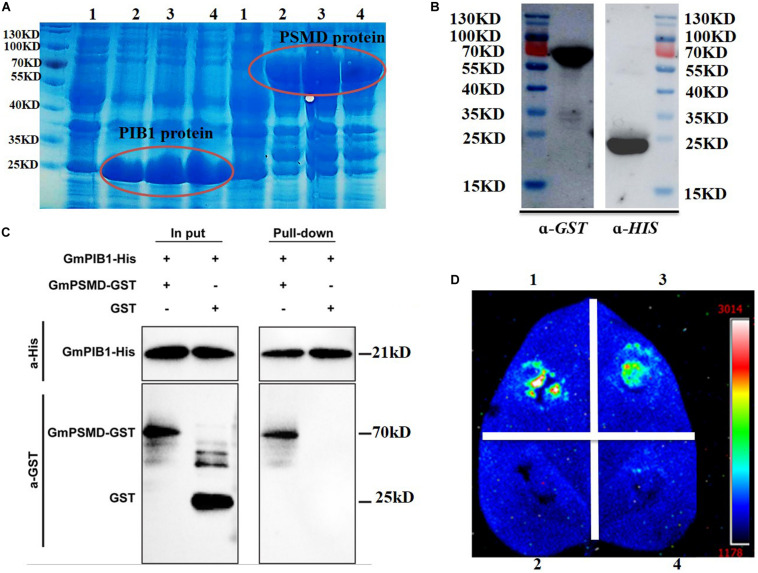
Verification of interaction between GmPIB1 and GmPSMD. **(A)** *Trans*etta cells containing pET29b*-GmPIB1* and pGEX-4T-1-*GmPSMD* recombinant plasmids were grown at 37°C for 1, 2, or 4 h with or without 0.5 mM isopropyl-β-D-thiogalactoside (IPTG) induction. Lane 1, *GmPIB1*-HIS and *GmPSMD*-GST proteins without IPTG induction; lane 2, GmPIB1-HIS and GmPSMD-GST proteins with IPTG induction for 1 h; lane 3, GmPIB1-HIS and GmPSMD-GST proteins with IPTG induction for 2 h; lane 4, GmPIB1-HIS and GmPSMD-GST proteins with IPTG induction for 4 h. **(B)** Immunoblotting of purified recombinant GmPIB1 with anti-His antibody and GmPSMD with anti-GST antibody. **(C)** Pull-down verification of GmPIB1 interaction with GmPSMD. **(D)** Verification of GmPIB1 interaction with GmPSMD by luciferase complementation. Transient expression of recombinant pCAMBIA1300-*GmPIB1*-cluc and pCAMBIA1300-*GmPSMD*-nluc plasmids after co-injection into *Nicotiana benthamiana*. 1, positive control GB-nLuc + FlS2-cluc; 2, negative control nluc; 3, GmPIB1-cLuc + GmPSMD-nluc; 4, negative control cluc.

We further verified the interaction between GmPIB1 and GmPSMD using pull-down assays *in vitro* and luciferase complementation assays *in vivo*. The expression levels of the fusion proteins GmPIB1-HIS and GmPSMD-GST were very high at 1, 2, and 4 h after IPTG induction (Figure 2A), indicating that the proteins were well purified and could be used for pull-down assays (Figure 2B). As shown by immunoblotting analysis, GmPSMD could interact with full-length GmPIB1 *in vitro* (Figure 2C). We then infiltrated *Agrobacterium* containing the recombinant plasmids pCAMBIA1300-*GmPIB1*-ccluc and pCAMBIA1300-*GmPSMD*-nluc into *Nicotiana benthamiana* leaves and observed uimioluminiscence at the infiltration areas after dark incubation for 2 days, and the results showed that GmPIB1 and GmPSMD can interact *in vivo* (Figure 2D).

### Bioinformatics Analysis of GmPSMD

**FIGURE 3:**
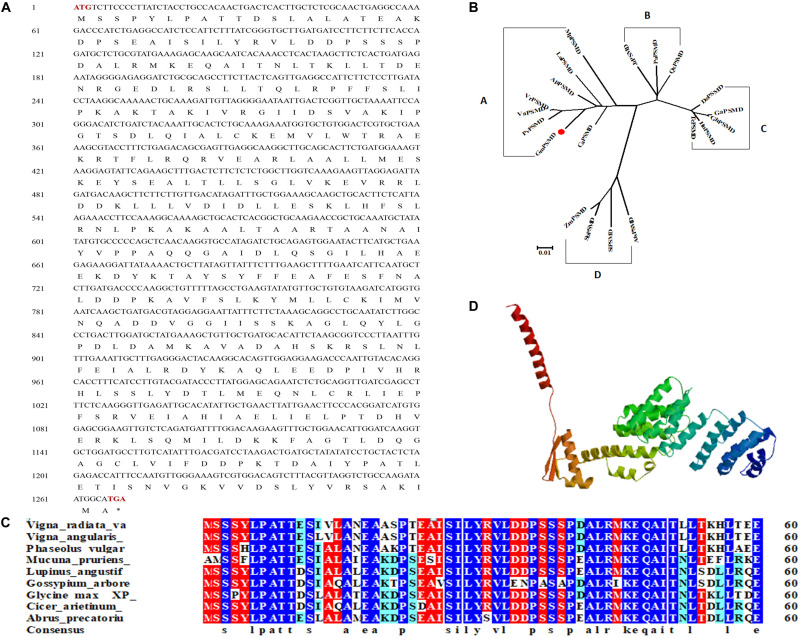
Sequence and structure of GmPSMD. **(A)** Nucleotide and amino acid sequences of GmPSMD cDNA. **(B)** Phylogenic analysis of GmPSMD and other PSMD proteins. GenBank accession numbers are as follows: *GmPSMD* (XP_014631720), *MpPSMD* (RDY13590), *LaPSMD* (XP_019417640), *ApPSMD* (XP_027354762), *VrPSMD* (XP_014501695), *VaPSMD* (XP_017409210), *CaPSMD (XP_012571699), PvPSMD (XP_007137150), JrPSMD (XP_018836824), PaPSMD* (XP_021807467), *QsPSMD* (XP_023895672), *SiPSMD* (XM_004963066.3), *SbPSMD* (XP_002446538), *AtPSMD* (XM_020338016.1), *ZmPSMD* (XM_020547787.1), *GaPSMD* (XP_017637832), *GhPSMD* (AXQ39585), *HuPSMD* (XP_021299831), *TMD* (EOY01828). **(C)** Alignment of the GmPSMD amino acid sequence with other sequences in subfamily A of PSMD. **(D)** Predicted three-dimensional structure of GmPSMD.

*GmPSMD* is located on chromosome 6 with a full length of 1,803 bp and a coding sequence of 1,269 bp; the encoded 423 amino acids contain a PCI domain belonging to the 26S proteasome 19S regulatory subunit (Figure 3A). Phylogenetic tree and alignment analyses revealed that PSMD is divided into four sub-families, A, B, C, and D, among which soybean *GmPSMD* and *Mucuna pruriens MpPSMD*, *Lupinus angustifolius LaPSMD*, *Abrus precatorius ApPSMD*, *Vigna radiata*. *VrPSMD*, *Phaseolus vulgaris PvPSMD*, *Vigna angularis VaPSMD*, and *Cicer arietinum CaPSMD* belong to subfamily A.

The amino acids of PSMD proteins from species belonging to the A subfamily in the phylogenetic tree were selected and analyzed by multi-column alignment using DNAMAN (Figure 3B). The sequence similarity of *GmPSMD* with *MpPSMD*, *LaPSMD*, *ApPSMD*, *VrPSMD*, *VaPSMD*, *CaPSMD*, and *PvPSMD* was 70.91, 94.55, 95.97, 95.97, 96.21, 94.55, and 95.02%, respectively (Figure 3C). *GmPSMD* had the highest similarity of 96.21% with *VaPSMD* and the lowest similarity of 70.91% with *MpPSMD*. The tertiary structure of GmPSMD was predicted using the website http://swissmodel.expasy.org. The protein consisted mainly of random coil, α-helix, β-sheet and extended long chains. There were 20 alpha helices and four beta folds (Figure 3D).

### GmPSMD Affects Soybean Resistance to *P. sojae*

**FIGURE 4:**
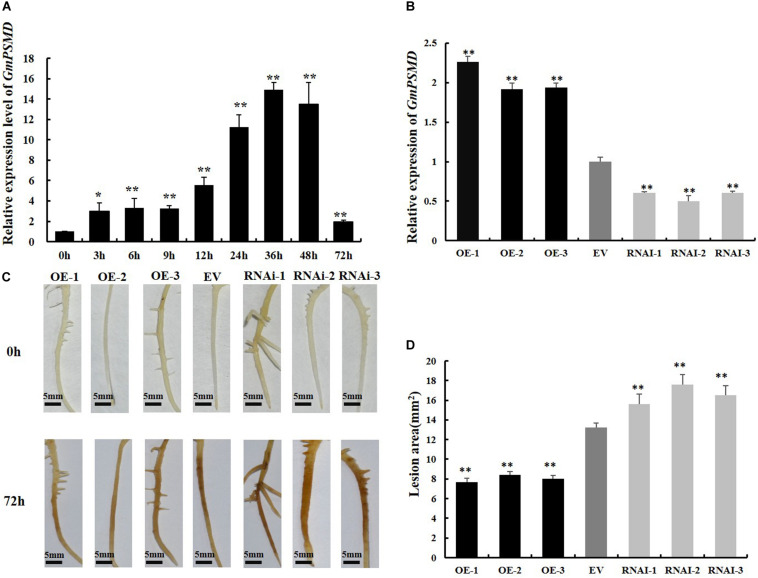
Resistance to *P. sojae* in *GmPSMD* transgenic hairy roots. **(A)** Relative expression levels of *GmPSMD* in soybean cultivar “Suinong 10” upon *P. sojae* infection, determined by qRT-PCR. Plants of the resistant cultivar “Suinong 10” were grown until the first true leaf was about to unfold and were inoculated with *P. sojae* zoospores. The leaves were sampled at 0, 3, 6, 9, 12, 24, 36, 48, and 72 h after inoculation with *P. sojae* race 1. **(B)** qRT-PCR analysis of the relative expression of *GmPSMD*-OE, *GmPSMD*-RNAi transgenic hair roots and EV. Test strips for detection of transgenic soybean hairy roots. **(C)** Typical infection phenotypes of EV (control), *GmPSMD*-RNAi and *GmPSMD*-OE (overexpressing) soybean hairy roots after *P. sojae* inoculation. The soybean hairy roots were cultured for about 2 weeks and were inoculated with *P. sojae* zoospores. bars, 5 mm. Hairy roots carrying empty vector (EV) were used as controls. **(D)** Lesion size measured from transgenic GmPSMD-OE, GmPSMD-RNAi transgenic hairy roots and EV at 72 h post-inoculation (hpi). The lesion size of each independent soybean line (*n* = 3) was calculated. Three biological replicates, each containing three technical replicates, were averaged and statistically analyzed by Student’s *t*-test (*P < 0.05; **P < 0.01). Bars show standard error of the mean.

To investigate whether *GmPSMD* is involved in the stress response induced by *P. sojae*, qRT-PCR was used to examine the transcript levels of *GmPSMD* in soybean cultivar “Suinong 10” infected with *P. sojae*. The relative expression of *GmPSMD* increased after inoculation with *P. sojae* race 1 and reached the highest level after 36 h (Figure 4A). This indicated that GmPSMD may be involved in the defense response against *P. sojae*.

To further explore the defense response of *GmPSMD* against *P. sojae*. *GmPSMD*-OE and *GmPSMD*-RNAi transgenic soybean hairy roots were obtained and the level of overexpression and silencing of *GmPSMD* transgenic soybean hairy roots were analyzed by qRT-PCR. The relative expression of *GmPSMD* in *GmPSMD*-OE transgenic soybean hairy roots was remarkably higher than that in control, while it showed lower level than control in *GmPSMD*-RNAi transgenic soybean hairy roots (Figure 4B). Characterization of disease resistance in *GmPSMD* transgenic soybean roots of the susceptible cultivar “Dongnong 50” revealed that the *GmPSMD*-OE soybean hairy roots turned slightly brown, while those transformed with pCAMBIA3301 empty vector and *GmPSMD*-RNAi showed rotting and browning of the inoculated parts (Figure 4C), and the lesion areas of *GmPSMD*-OE transgenic soybean hairy roots were significantly smaller than that in the control, while the GmPSMD-RNAi soybean hairy roots were significantly larger than that in control (Figure 4D).

**FIGURE 5:**
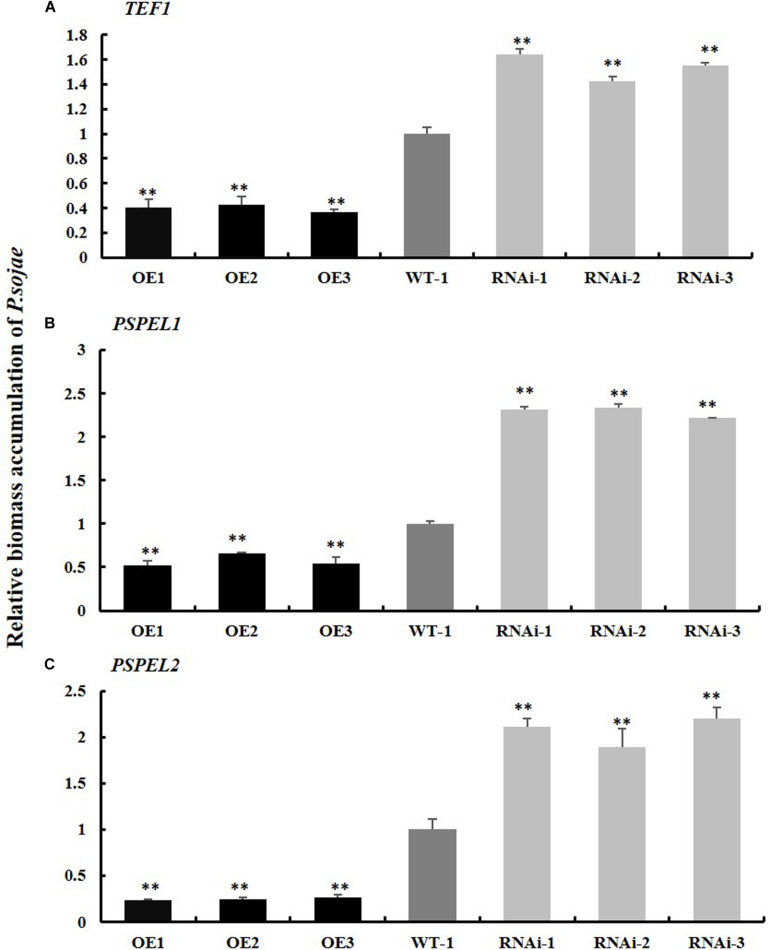
Relative biomass accumulation of *GmPSMD* in transgenic soybean hairy roots. **(A–C)** The transcript levels of TEF1, PSPEL1, and PSPEL2. Quantitative reverse transcription-polymerase chain reaction (RT-PCR) analysis of the relative biomass of *P. sojae* in *GmPSMD*-OE, *GmPSMD*-RNAi transgenic hair roots and EV based on *P. sojae TEF1*, *PSPEL1*, and *PSPEL2* transcript levels. Three biological replicates, each containing three technical replicates, were averaged and statistically analyzed by Student’s *t*-test (^∗^*P* < 0.05; ^∗∗^*P* < 0.01). Bars show standard error of the mean.

Moreover, the relative biomass of *P. sojae* based on transcript levels of the *P. sojae TEF1* (GenBank accession no. EU079791), *PSEL1* (GenBank accession no. CF840149), and *PSEL2* (GenBank accession no. CF839332) in infected soybean hairy roots after 2 days of incubation with zoospores of *P. sojae* was significantly (^∗∗^*P* < 0.01) lower in *GmPSMD*-OE lines than in EV hairy roots. Conversely, the biomass of *P. sojae* in *GmPSMD*-RNAi roots was higher than that in the control (Figures 5A–C). These results indicated that overexpression of *GmPSMD* can improve the resistance of soybean hairy roots to *P. sojae*, while *GmPSMD*-RNAi transgenic soybean hairy roots showed increased susceptibility to *P. sojae.*

### GmPSMD May Affect Soybean Resistance to *P. sojae* by Reducing the Level of ROS

**FIGURE 6:**
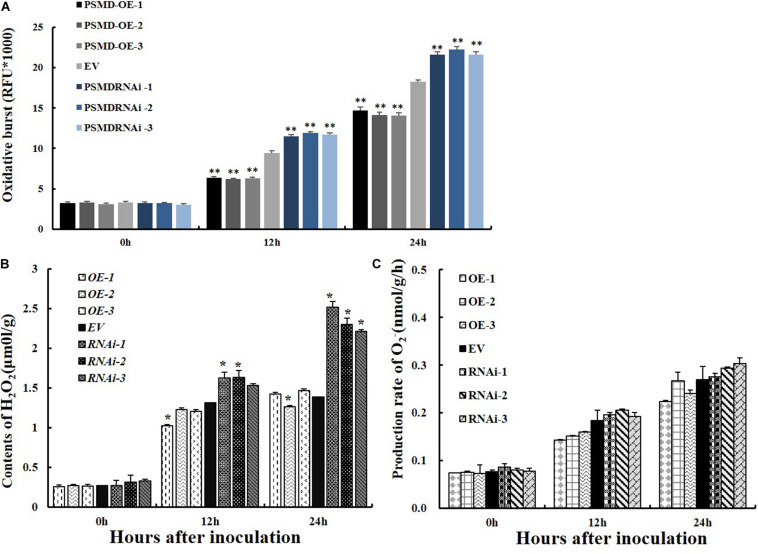
Analysis of ROS, H_2_O_2_ and O_2_^–^ relative contents and 26S proteasome activity in transgenic and EV soybean hairy roots. **(A)** Relative contents of total ROS in EV, *GmPSMD*-OE-1, *GmPSMD*-OE-2, *GmPSMD*-OE-3, *GmPSMD*-RNAi-1, *GmPSMD*-RNAi-2, and *GmPSMD*-RNAi-3 soybean hairy roots at 0, 12 and 24 h after *P. sojae* infection. **(B,C)** Relative contents of H_2_O_2_ and O_2_^–^ in EV, *GmPSMD*-OE-1, *GmPSMD*-OE-2, *GmPSMD*-OE-3, *GmPSMD*-RNAi-1, *GmPSMD*-RNAi-2, and *GmPSMD*-RNAi-3 soybean hairy roots at 0, 12, and 24 h after *P. sojae* infection. The soybean hairy roots were cultured for about 2 weeks and were inoculated with *P. sojae* zoospores. Three biological replicates, each containing three technical replicates, were averaged and statistically analyzed by Student’s *t*-test (*P < *0.05*; **P < 0.01). Bars show standard error of the mean.

ROS are key signaling molecules in the interaction between plants and pathogens under stress (Mittler et al., 2004; Shigeoka and Maruta, 2014). To determine if GmPSMD affects the resistance of soybean hairy roots to *P. sojae* by affecting the production of ROS, the relative ROS levels in *GmPSMD*-RNAi, *GmPSMD*-OE, and EV transgenic soybean hairy roots were analyzed at 0, 12, and 24 h after inoculation with *P. sojae*. Accumulation of ROS in *GmPSMD*-RNAi hairy roots reached significant levels after 24 h compared with that in control, while ROS accumulation in *GmPSMD*-OE hairy roots was lower than that in control (Figure 6A).

**FIGURE 7:**
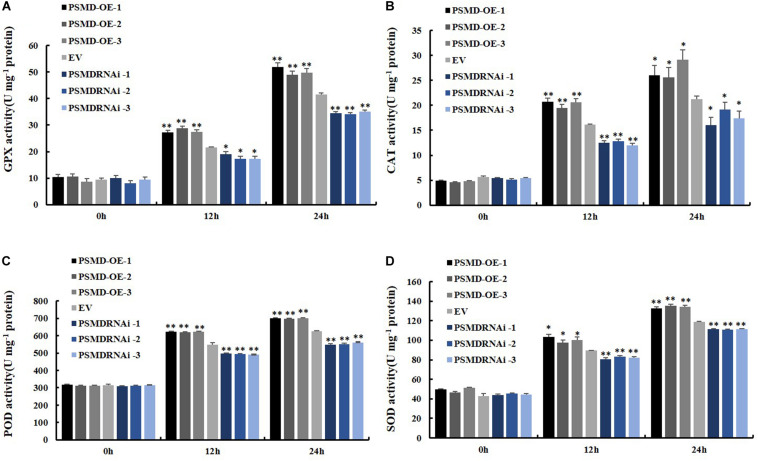
Determination of antioxidant enzyme activity. **(A)** Determination of antioxidant enzyme activity of GPX. **(B)** Determination of antioxidant enzyme activity of CAT. **(C)** Determination of antioxidant enzyme activity of POD. **(D)** Determination of antioxidant enzyme activity of SOD. The soybean hairy roots were cultured for about 2 weeks and were inoculated with *P. sojae* zoospores. Three biological replicates, each containing three technical replicates, were averaged and statistically analyzed by Student’s *t*-test (*P < 0.05; **P < 0.01). Bars show standard error of the mean.

ROS include hydrogen peroxide (H_2_O_2_) and superoxide anions (O_2_^–^). Therefore, the contents of H_2_O_2_ and O_2_^–^ were further analyzed after inoculation with *P. sojae* in EV, *GmPSMD*-OE, and *GmPSMD*-RNAi transgenic soybean hairy roots. The relative H_2_O_2_ levels in all three transgenic soybean hairy roots increased gradually with the extension of inoculation time. However, the accumulation of H_2_O_2_ in *GmPSMD*-OE transgenic soybean hairy roots was lower than that in the control, while H_2_O_2_ accumulation in transgenic soybean hairy roots of *GmPSMD*-RNAi was higher than that in the control (Figure 6B). Levels of O_2_^–^ in transgenic soybean hairy roots were not remarkably different from those of the control (Figure 6C). SOD, POD, CAT and GPX as the main antioxidant enzymes participated in the scavenging of reactive oxygen species and alleviated the damage of membrance system (Zhang et al., 2008). The antioxidant enzymes activity of SOD, POD, CAT, and GPX were analyzed after inoculation with *P. sojae* in EV, *GmPSMD*-OE, and *GmPSMD*-RNAi transgenic soybean hairy roots. The antioxidant enzymes activity increased gradually with the extension of inoculation time. However, the antioxidant enzymes activity in *GmPSMD*-OE transgenic soybean hairy roots was higher than that in control, while the antioxidant enzymes activity in transgenic soybean hairy roots of *GmPSMD*-RNAi was lower than that in the control (Figure 7).

### GmPSMD May Affect 26S Proteasome Activity in GmPSMD and GmPIB1 Transgenic Soybean Hairy Roots During *P. sojae* Infection

**FIGURE 8:**
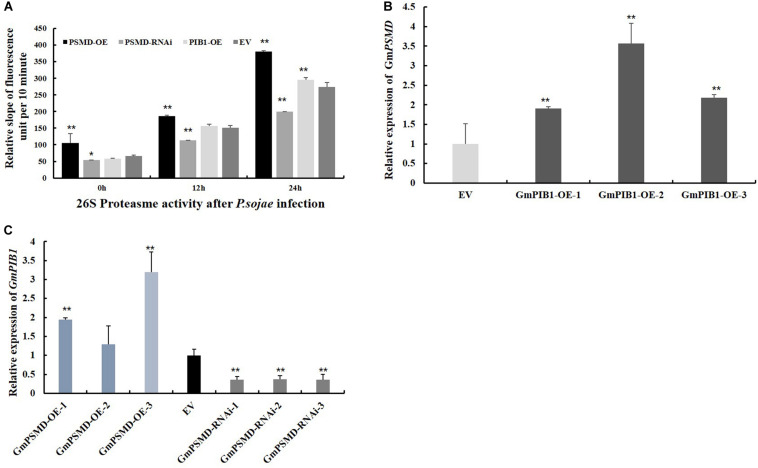
26S proteasome activity and the transcript levels of *GmPIB1* and *GmPSMD* in transgenic and EV soybean hairy roots. **(A)** The 26S proteasome activity in *GmPSMD*-OE, *GmPSMD*-RNAi and *GmPIB1*-OE transgenic and EV soybean hairy roots inoculated with *P. soaje* was determined. The soybean hairy roots were cultured for about 2 weeks and were inoculated with *P. sojae* zoospores. The slope of fluorescence units per 10 min represents the activity of 26S proteasome. The proteasome substrate Ø, fluorogenic was used as fluorescent substrate. Fluorescence was detected at 460 nm emission wavelength and 380 nm excitation wavelength at 25°C. The changes of fluorescence units in 10 min were recorded. Three biological replicates, each containing three technical replicates, were averaged and statistically analyzed by Student’s *t*-test (*P < 0.05; **P < 0.01). Bars show standard error of the mean. The values on the bar chart are the average of three biological repetitions. **(B)** qRT-PCR analysis of the relative expression of *GmPSMD* in *GmPIB1*-OE and EV soybean hairy roots based on EF1βtranscript levels. **(C)** qRT-PCR analysis of the relative expression of *GmPIB1* in *GmPSMD*-OE, *GmPSMD*-RNAi, and EV soybean hairy roots based on *EF1*β transcript levels. Three biological replicates, each containing three technical replicates, were averaged and statistically analyzed by Student’s *t*-test (*P < 0.05; **P < 0.01). Bars show standard error of the mean.

To verify if GmPSMD affects 26S proteasome activity of transgenic soybean hairy roots and explore its role in response to *P. sojae* infection. The 26S proteasome activity was measured in *GmPSMD*-OE, *GmPSMD*-RNAi, *GmPIB1*-OE, and EV transgenic soybean hairy roots inoculated with *P. sojae*. The results showed that the 26S proteasome activity in hairy roots of *GmPSMD*-OE and *GmPIB1*-OE was remarkably higher than that in control, while the 26S proteasome activity in *GmPSMD*-RNAi soybean hairy roots was remarkably lower than that in the control after infection with *P. sojae* (Figure 8A). These results indicate that *GmPSMD* could increase the 26S proteasome activity in transgenic soybean hairy roots during *P. sojae* infection. We also found that the expression levels of *GmPIB1* in *GmPSMD*-OE transgenic soybean hairy roots and GmPSMD in *GmPIB1*-OE transgenic soybean hairy roots increased, respectively, which proved that they were a pair of positive regulatory factors (Figures 8B,C).

## Discussion

Complex plant innate immune system networks have gradually developed with the evolution of plants and pathogens, and include the pathogen-associated molecular pattern-triggered immune response caused by pathogen-related molecular models and the effector-triggered immune response (Jones and Dangl, 2006). Identification of genes related to *P. sojae* infection has helped our understanding of the genetic mechanism of the response against Phytophthora root and stem rot of soybean (Xu et al., 2014; Cheng et al., 2015; Kong et al., 2015; Zhang et al., 2017). The bHLH transcription factor GmPIB1 is a positive regulator that responds to *P. sojae* infection (Cheng et al., 2018).

In this study, we identified the GmPSMD protein interacting with GmPIB1 using mass spectrometry. GmPSMD is a component of the 26S proteasome regulatory subunits. Several studies have demonstrated that the 26S proteasome is involved in the resistance of rice and Arabidopsis to pathogens (Qiao et al., 2004; Park et al., 2012; Hatsugai et al., 2015). The 26S proteasome subunits can interact with pathogens effectors such as HopZ4, AvrPiz-t to inhibit their own activity and trigger PTI and ETI responses (Park et al., 2012; Üstün et al., 2014). They can also degrade ubiquitinated modified proteins in the ubiquitin/26S proteasome pathway to participate in disease response (Luo et al., 2010; Liu et al., 2015). Although the 26S proteasome is widely involved in the resistance response of plants to pathogens, little is known about its involvement in the defense response to *P. sojae*. As GmPIB1 is a positive regulator of this response, it is likely that GmPSMD also participates and affects the resistance of soybean to *P. sojae*. Expression of *GmPSMD* in soybean was significantly increased after infection by *P. sojae*, indicating that GmPSMD might participate in the process of soybean resistance to *P. sojae* (Figure 4A). Furthermore, overexpression of *GmPSMD* significantly improved soybean resistance to *P. sojae*, while GmPSMD-RNAi produced the opposite symptoms (Figure 4C). The biomass accumulation of *P. sojae* in *GmPSMD*-RNAi transgenic soybean hairy roots was higher than that in controls byqRT-PCR (Figure 5), further proving that GmPSMD regulates soybean resistance to *P. sojae*. These findings suggest that GmPSMD plays a significant role in defense against *P. sojae* in soybean hairy roots.

ROS are important signaling molecules regulating plant responses to biological stress, including the process of plant–pathogen interaction (Hückelhoven and Kogel, 2003). They are produced not only by primary metabolism, but also by apoplast-localized oxidases or peroxidases and plasma membrane (Suzuki et al., 2011; Cheng et al., 2015; Zhang et al., 2015, 2016). Evidence for the role of ROS during attack by microbial pathogens was provided by inhibition of ROS accumulation and plant defense by chemicals like diphenylene iodonium chloride which is thought to suppress a ROS-producing NADPH oxidase (Jabs et al., 1997). GmPIB1 is a positive regulatory factor in the response to *P. sojae* infestation, and ROS levels in *GmPIB1*-OE, *GmPIB1*-RNAi and EV transgenic soybean hairy roots indicated that GmPIB1 affects soybean resistance to *P. sojae* through regulating ROS levels (Cheng et al., 2018). We therefore proposed that GmPSMD might improve the resistance of transgenic soybean hairy roots to *P. sojae* by affecting levels of ROS and determined the levels of ROS in EV, *GmPSMD*-OE and *GmPSMD*-RNAi transgenic soybean hairy roots. The results indicated that *GmPSMD* suppresses ROS accumulation (Figure 6A), which is consistent with our expectation.

Hydrogen peroxide (H_2_O_2_) and superoxide anion (O_2_^–^) are the main components of ROS. Under stress, accumulation of H_2_O_2_ in plant cells kills pathogens and induces immune reactions. Meanwhile, lower concentrations of H_2_O_2_ can also act as signaling molecules to induce a series of genes encoding defense-reactive protein, increasing resistance to pathogens (Foyer and Shigeoka, 2011). We further analyzed levels of H_2_O_2_ and O_2_^–^ using qRT-PCR. The results showed that *GmPSMD* could reduce H_2_O_2_ accumulation (Figure 6B). However, it’s remarkable that there was no obvious difference in the level of O_2_^–^ between transgenic soybean hairy roots and the control (Figure 6C).

Antioxidant enzymes in plants are able to remove part of the reactive oxygen species in time and maintain the oxygen balance (Noctor and Foyer, 1998; Mittler et al., 2004). SOD, POD, CAT, and GPX as the main antioxidant enzymes participated in the scavenging of reactive oxygen species and alleviated the damage of membrance system (Zhang et al., 2008). The enzymes activity of SOD, POD, CAT, and GPX were further analyzed, and the results showed that GmPSMD could increase enzymes activity of SOD, POD, CAT, and GPX (Figure 7). Moreover, the activities of 26S proteasome increased significantly in GmPSMD-OE and GmPIB1-OE transgenic soybean hairy roots comparing with that in control after infection with *P. sojae* (Figure 8A), which means that the activities of 26S proteasome might be critical for GmPSMD and GmPIB1 in response to *P. sojae*. In addition, we found that the expression levels of GmPIB1 in *GmPSMD*-OE transgenic soybean hairy roots and *GmPSMD* in *GmPIB1*-OE transgenic soybean hairy roots increased, respectively (Figures 8B,C), which proved that they were a pair of positive regulatory factors. In this study, both *GmPIB1* and *GmPSMD* can improve the resistance of soybean hairy roots to *P. sojae* by decreasing the production of ROS. The difference is that *GmPIB1* inhibits the expression of *GmSPOD1*, while *GmPSMD* influences the activity of antioxidant enzymes and inhibit the accumulation of ROS. Taken together, these data indicate that GmPSMD could improve resistance to *P. sojae* in soybean by improving antioxidant enzymes activity of CAT, POD, SOD, and GPX to reduce levels of ROS.

## Data Availability Statement

All datasets generated for this study are included in the article/Supplementary Material.

## Author Contributions

PX and SZ designed the experiments. TL, HW, ZL, and ZP performed the experiments. CZ, MZ, BN, BS, SL, ZH, WW, YL, and JW analyzed the data. TL, PX, and SZ wrote the manuscript. All authors contributed to the article and approved the submitted version.

## Conflict of Interest

The authors declare that the research was conducted in the absence of any commercial or financial relationships that could be construed as a potential conflict of interest.

## Footnotes

1 http://www.lynnon.com/

2 http://www.sbg.bio.ic.ac.uk/phyre2

## Supplementary Material

Supplementary Figure 1 **(A)** Construction of pCAMBIA3301-GmPIB1 recombinant vector. **(B)** Immunoblotting analysis of *GmPIB1*-OE transgenic soybean hairy roots with hairy roots carrying empty vector (EV) as control. Total protein extracts were analyzed in a 12% SDS-PAGE gel and probed with an anti-Myc antibody. **(C)** Test strips for detection of *GmPSMD* transgenic soybean hairy roots.

Supplementary Figure 2 Preliminary interaction verification of GmPIB1 with candidate proteins in yeast cells.

Supplementary Table 1 Primer sequences used in this study.

Supplementary Table 2 Candidate genes from mass spectrum data.
