# Supplementary figures and images for: The 26S Proteasome Regulatory Subunit GmPSMD Promotes Resistance to Phytophthora sojae in Soybean
Source: Front Plant Sci. 2021 Jan 28;12:513388. doi: 10.3389/fpls.2021.513388 (PMC7876454; doi:10.3389/fpls.2021.513388)

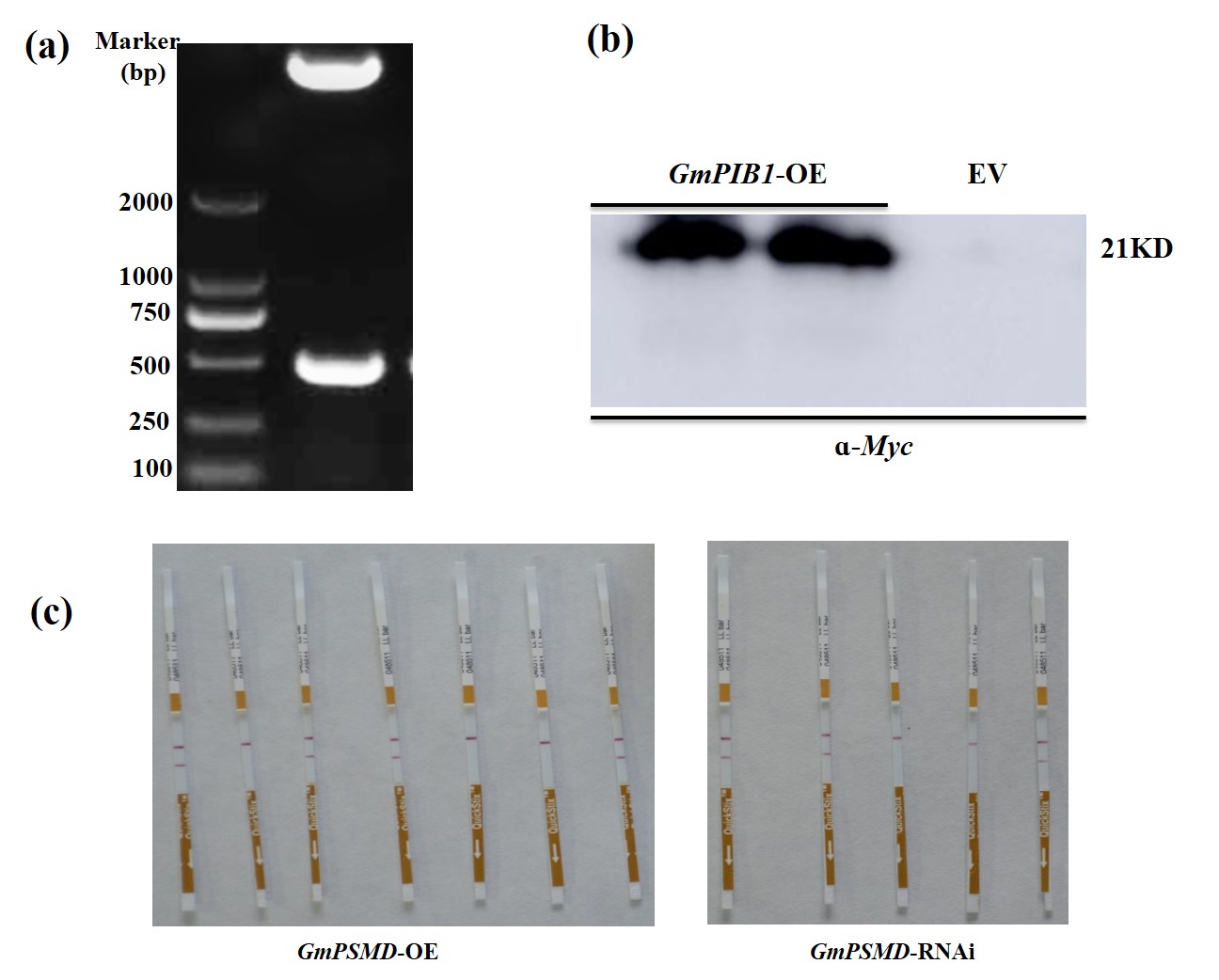

Supplement: Supplementary file 1 [file Image_1.JPEG]

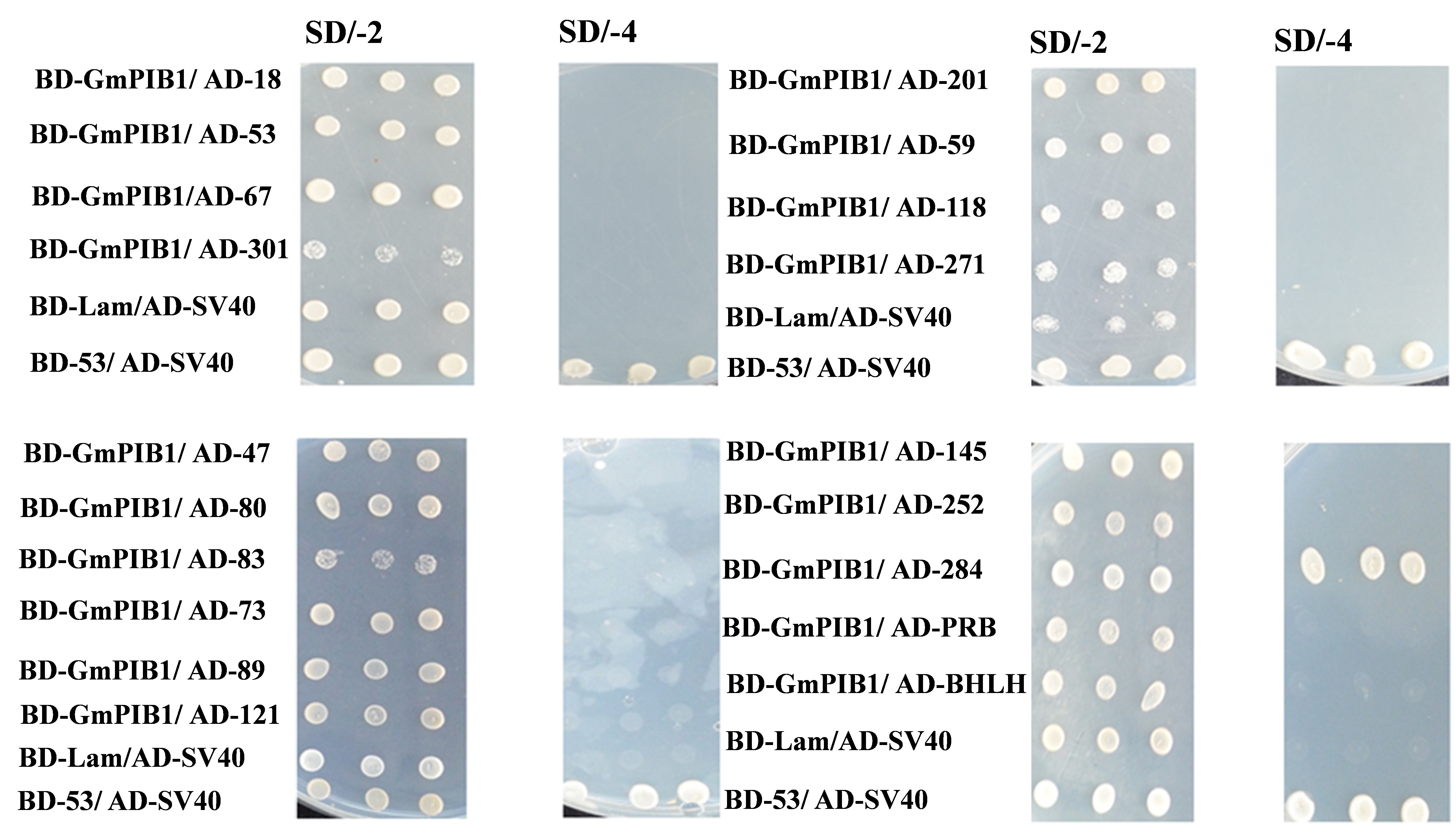

Supplement: Supplementary file 2 [file Image_2.JPEG]

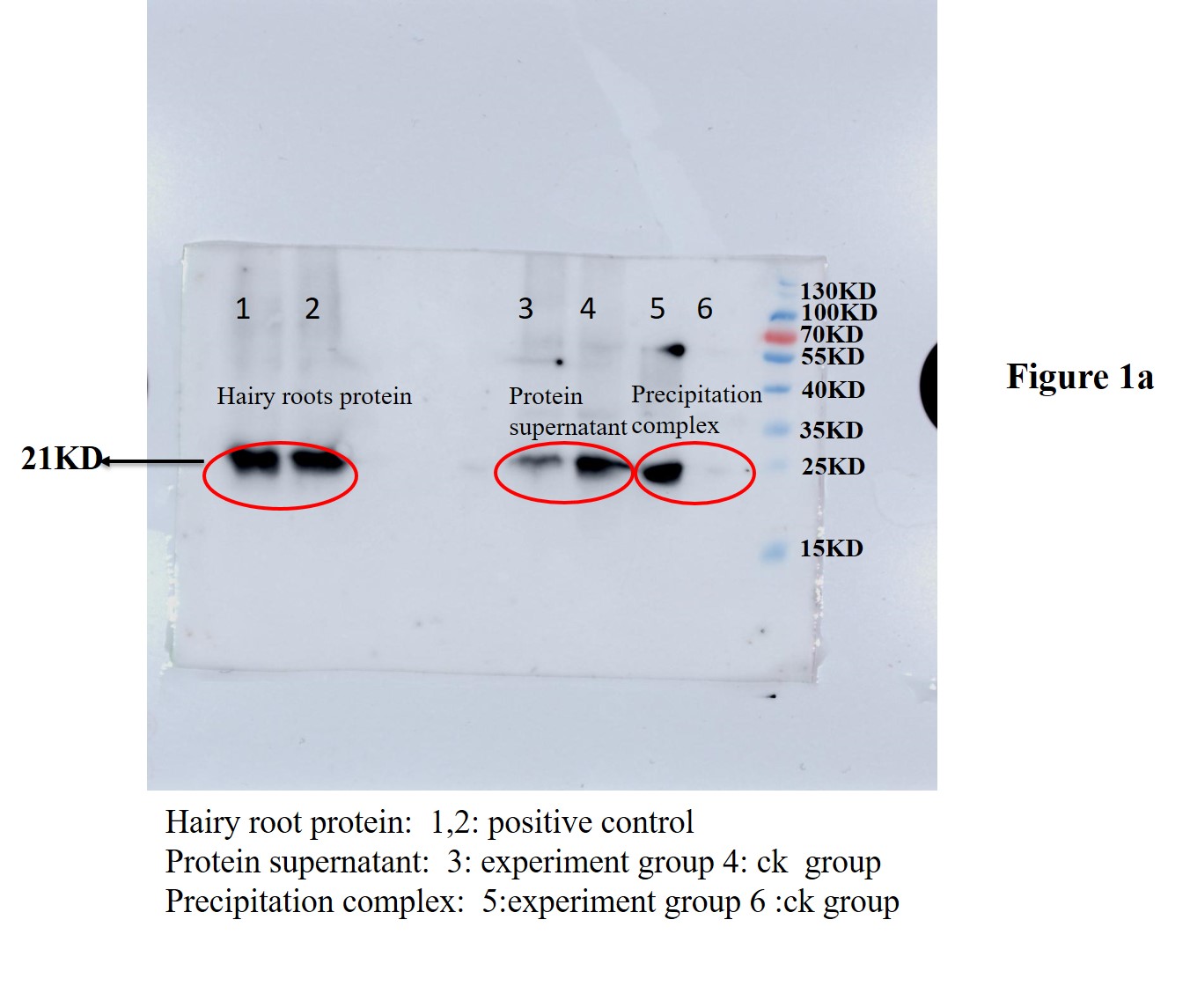

Supplement: Supplementary Figure 1 — (A) Construction of pCAMBIA3301-GmPIB1 recombinant vector. (B) Immunoblotting analysis of GmPIB1-OE transgenic soybean hairy roots with hairy roots carrying empty vector (EV) as control. Total protein extracts were analyzed in a 12% SDS-PAGE gel and probed with an anti-Myc antibody. (C) Test strips for detection of GmPSMD transgenic soybean hairy roots. [file Image_3.JPEG]

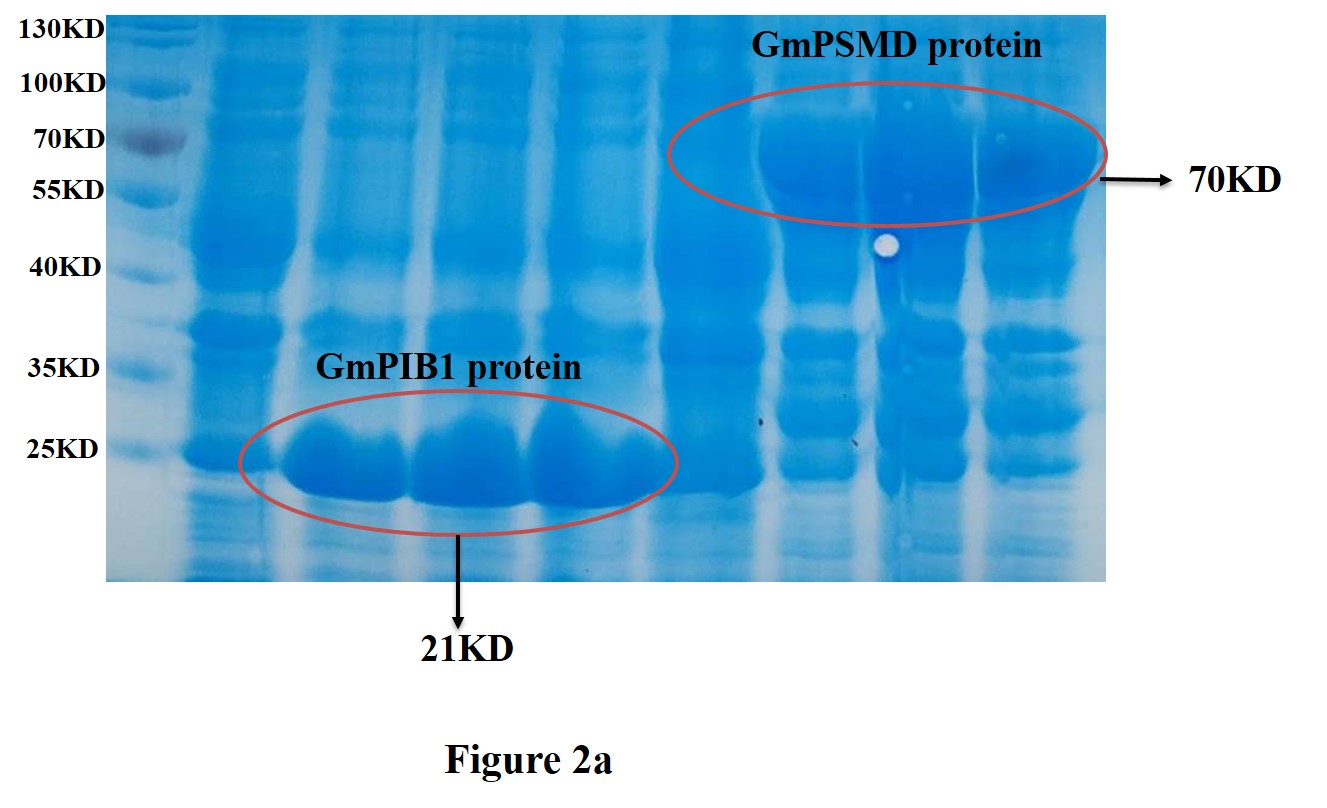

Supplement: Supplementary Figure 2 — Preliminary interaction verification of GmPIB1 with candidate proteins in yeast cells. [file Image_4.JPEG]

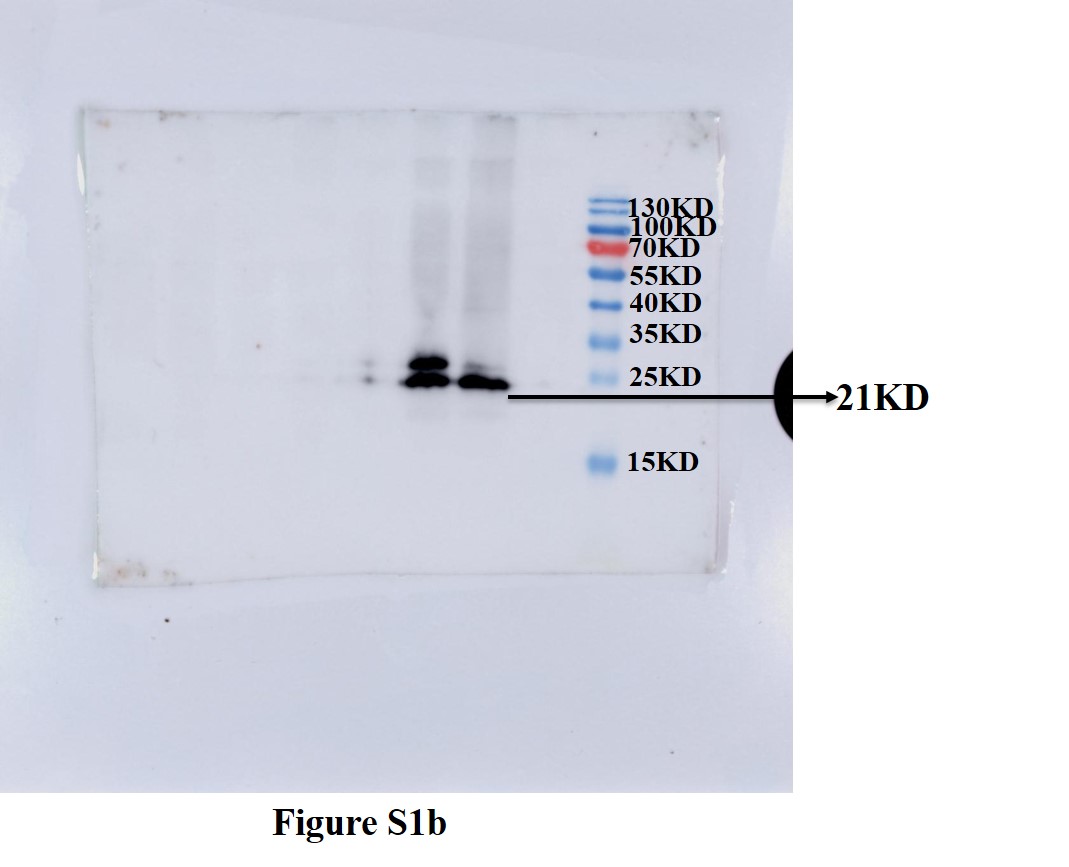

Supplement: Supplementary file 5 [file Image_5.JPEG]
