# Supplementary material for: The 26S Proteasome Regulatory Subunit GmPSMD Promotes Resistance to Phytophthora sojae in Soybean
Source: Front Plant Sci. 2021 Jan 28;12:513388. doi: 10.3389/fpls.2021.513388 (PMC7876454; doi:10.3389/fpls.2021.513388)
Supplement: Supplementary Table 1 — Primer sequences used in this study. [file Data_Sheet_1.docx]

**Table S1.** Primer sequences used in this study.

**Primers for quantitative real-time PCR**

| **Primer name** | **Primers (5’-3’)** |
| --- | --- |
| *PSMD-qPCR-F*  *PSMD-qPCR-R*  *SPOD-qPCR-F*  *SPOD-qPCR-R*  *SOD-qPCR-F*  *SOD-qPCR-R*  *EF1β-F*  *EFIβ-R*  *PSEL1-F:*  *PSEL1-R:*  *PSEL2-F:*  *PSEL2-R:* | GTCTTCCCCTTATCTACCTGCC  CCTCTCCCCTATTCTCATCAGTG  TTCGCAAAGTGTTGTAGTTCTC  AGACGGTGACGAGAAGCAAC  CGTGCTGCGATAACAAGGAT  AACCACTGCCAACCAGGACC  CCACTGCTGAAGAAGATGATGATG  AAGGACAGAAGACTTGCCACTC CCGCGTACGTGGCTTTGGTGAG  ATCTTGGCGACTGAGGCTGCTTAC  CCCACGCCAAACTCCACGAT  CGTCCGTGGTCTCGGTGCTG |

**Primers for constructs in plant transformation**

| **Primer name** | **Primers (5’-3’)** |
| --- | --- |
| *GmPSMD-MycF*  *GmPSMD*-*MycR*  Gm*PSMDRNAi1-F*  Gm*PSMDRNAi1-R*  Gm*PSMDRNAi2-F*  Gm*PSMDRNAi2-R* | AGAGATAGATTTGTAGAGCCATGGTGAT  GTCTTCCCCTTATCTACCTG  ACCGTTAATTAACCCCACGTGATATGCCATTATCTTGGCAGA  CTCGAGATGTCTTCCCCTTATCTACCTGAGT  CCATGGTTGCTTGCTCTTTCATACGCAG  TCTAGAATGTCTTCCCCTTATCTACCTG  GGATCCTTGCTTGCTCTTTCATACGCA |

**Primers for Prokaryotic Expression**

| **Primer name** | **Primers (5’-3’)** |
| --- | --- |
| *GmPSMD*-GSTF  *GmPSMD-*GSTR  *GmPIB1-HISF*  *GmPIB1-HISR* | ATCGGATCTGGTTCCGCGTGGATCCATGTCTTCCCCTTATCTACCTG  CCGCTCGAGTCGACCCGGGAATTCATATGCCATTATCTTGGCAGA  GGAATTCCATGGATTCTAGGCGGCGT  GCTCGAGATCCATGTTTTGGATTGCTTG |

**Primers for Luciferase Complementary assays**

| **Primer name** | **Primers (5’-3’)** |
| --- | --- |
| *GmPIB1-*CclucF*GmPIB1*-CclucR *GmPSMD-NlucFGmPSMD-NlucR* | ACACGGGGGACGAGCTCATGGATTCTAGGCGGCGT  TACGAGATCTGGTCGACATCCATGTTTTGGATTGCTTG  ACACGGGGGACGAGCTCATGTCTTCCCCTTATCTACCTG  TCGTATGGGTAGTCGACTGCCATTATCTTGGCAGAC |

**Primers for Yeast two-hybrid assays**

| **Primer name** | **Primers (5’-3’)** |
| --- | --- |
| *GmPSMD-YF*  *GmPSMD-YR* | GCCATGGAGGCCAGTGAATTCATGGGTGCCTACAAGTATGTTT TGCAGCTCGAGCTCGATGGATCCATTAGCGGTAACGGCGGA |

**Table S2.**Candidate genes from mass spectrum data

| **Number** | **Accession** | **Description** |
| --- | --- | --- |
| 18 | A0A0R0JYS9 | Peroxidase |
| 53 | D4N5G3 | Rubisco activas |
| 67 | I1KV08 | Uncharacterized protein |
| 73 | A0A0R4J2Q1 | Uncharacterized protein |
| 80 | I1K7H0 | Uncharacterized protein |
| 83 | I1LKM5 | Uncharacterized protein |
| 89 | I1JE14 | GlycosyLtransferase |
| 121 | I1KYY4 | 40S ribosomal protein |
| 103 | A0A0R0GZF0 | Annexin |
| 145 | A0A0R0JSG7 | Peroxidase |
| 173 | I1KGP0 | Uncharacterized protein |
| 269 | I1NF03 | Uncharacterized protein |
| 270 | I1K3S5 | Adenosylhomocysteinase |
| 273 | I1N1W7 | Uncharacterized protein |
| 284 | I1K970 | 26S proteasome regulatory subunit |
| 289 | Q2I0H4 | GlyceraLdehyde-3-phosphate dehydrogenase |
| 301  33 | A0A0R0IRH4 | Phosphoglycerate kinase |
| prb | Glyma.14G043700 | Glucuronosyl Transferases |
| bhlh | XR_003264399.1 | *Glycine max* 28S ribosomal RNA |
